# Chiral transmission by an open evolution trajectory in a non-Hermitian system

**DOI:** 10.1038/s41377-024-01409-1

**Published:** 2024-03-05

**Authors:** Xiaoqian Shu, Qi Zhong, Kai Hong, Oubo You, Jian Wang, Guangwei Hu, Andrea Alù, Shuang Zhang, Demetrios N. Christodoulides, Lin Chen

**Affiliations:** 1 Wuhan National Laboratory for Optoelectronics and School of Optical and Electronic Information, Huazhong University of Science and Technology, Wuhan, 430074 China; 2 Zhejiang Lab, Hangzhou, 311121 China; 3 CREOL, College of Optics and Photonics, University of Central Florida, Orlando, Florida 32816 USA; 4 Department of Physics, The University of Hong Kong, Hong Kong, China; 5 School of Electrical and Electronic Engineering, Nanyang Technological University, 50 Nanyang Avenue, Singapore, 639798 Singapore; 6 Photonics Initiative, Advanced Science Research Center, City University of New York, New York, NY 10031 USA; 7 Shenzhen Huazhong University of Science and Technology Research Institute, Shenzhen, 518063 China

**Keywords:** Optical physics, Optics and photonics

## Abstract

Exceptional points (EPs), at which two or more eigenvalues and eigenstates of a resonant system coalesce, are associated with non-Hermitian Hamiltonians with gain and/or loss elements. Dynamic encircling of EPs has received significant interest in recent years, as it has been shown to lead to highly nontrivial phenomena, such as chiral transmission in which the final state of the system depends on the encircling handedness. Previously, chiral transmission for a pair of eigenmodes has been realized by establishing a closed dynamical trajectory in parity-time- (PT-) or anti-PT-symmetric systems. Although chiral transmission of symmetry-broken modes, more accessible in practical photonic integrated circuits, has been realized by establishing a closed trajectory encircling EPs in anti-PT-symmetric systems, the demonstrated transmission efficiency is very low due to path-dependent losses. Here, we demonstrate chiral dynamics in a coupled waveguide system that does not require a closed trajectory. Specifically, we explore an open trajectory linking two infinite points having the same asymptotic eigenmodes (not modes in PT- and anti-PT-symmetric systems), demonstrating that this platform enables high-efficiency chiral transmission, with each eigenmode localized in a single waveguide. This concept is experimentally implemented in a coupled silicon waveguide system at telecommunication wavelengths. Our work provides a new evolution strategy for chiral dynamics with superior performance, laying the foundation for the development of practical chiral-transmission devices.

## Abstract (web summary)

We explore an open trajectory linking two infinite points having the same asymptotic eigenmodes, demonstrating that this platform enables high-efficiency chiral transmission, with each eigenmode localized in a single waveguide.

## Graphical abstract

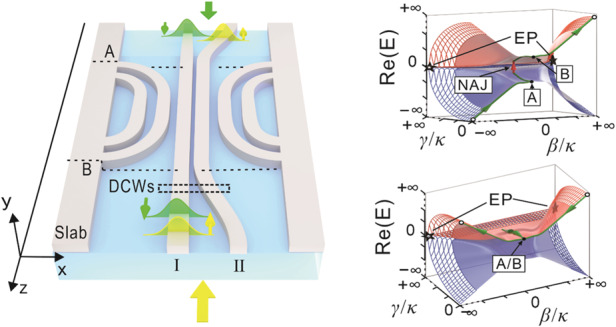

## Introduction

Exceptional points (EPs) are singularities where the eigenvalues and eigenstates of the Hamiltonian of a non-Hermitian system simultaneously coalesce, which exhibit many intriguing effects and phenomena in various physical disciplines^1–6^, including electronics and photonics. By tailoring the distribution of gain and loss, a variety of photonic systems, such as microcavities^7–10^, coupled waveguides^11–19^, gratings^20,21^ and photonic crystals^22,23^, have been utilized to study EPs and the associated unconventional physics. The unique features of EPs have enabled the observation of various intriguing phenomena, including loss-induced transmission enhancement^11^, enhanced sensitivity^24–27^, unidirectional invisibility^28,29^, optical limiters^30^, and single-mode lasing^8,31^, which not only are of importance from the fundamental standpoint, but also enable novel optical devices and technologies.

Among various exotic phenomena, encircling EPs in a non-Hermitian system by varying parameters of the system has inspired growing research interest due to its intrinsic chiral dynamics, in which the final state of the system depends on the encircling handedness^14,32–35^. This chiral behavior has been extensively demonstrated by mapping the required Hamiltonian onto coupled waveguides, leading to the observation of asymmetric mode switching^13,15,17,18,20,21,36–38^. Counterintuitively, it has been proven that chiral state conversion can be achieved through an EP-excluding cycle in a non-Hermitian system^21,33,34,39^. For the evolution strategy in PT-symmetric systems, the Hamiltonian evolution should follow a closed trajectory winding around EPs, such that the system has the same Hamiltonian and eigenstates at the starting and ending points. The chiral conversion usually occurs between two modes that correspond to symmetrical and anti-symmetrical waveguide modes, respectively, but the conversion efficiency is typically low due to path-dependent losses^13,15,20,36^. To overcome this limitation, it was recently proposed that Hamiltonian hopping and fast encirclement of EPs could boost the transmission efficiency to near unity^37,38^. Chiral transmission of symmetry-broken modes has been recently demonstrated by establishing a closed trajectory encircling EPs in anti-PT-symmetric systems, but it again suffers from very low transmission efficiency (around 4% in an experiment at microwave frequencies)^16^.

In this work, we report the theoretical and experimental demonstration of chiral conversion between modes localized in individual waveguides. Our work goes beyond the previously adopted closed evolution trajectory winding around EPs by demonstrating an open evolution trajectory that takes advantage of the same asymptotic modes at infinite points (not symmetrical/anti-symmetrical modes in PT- and symmetry-broken modes in anti-PT-symmetric systems). In such a dynamical non-Hermitian system, non-adiabatic jumps (NAJs), - the key factor for inducing a chiral response - originate from coupling loss selectively to one of the eigenmodes during the evolution. The chiral dynamics is theoretically predicted and experimentally demonstrated in coupled silicon waveguides. The resultant chiral mode converters can localize optical energy within a single waveguide with high-efficiency transmission. The chiral mode converters based on fast encirclement of EPs need three-step electron-beam lithography (EBL) and precise alignment when forming the Cr layer on the silicon waveguides^38^. In contrast, the chiral mode converters based on open evolution trajectories can relax the fabrication requirements.

## Results

**Fig. 1:**
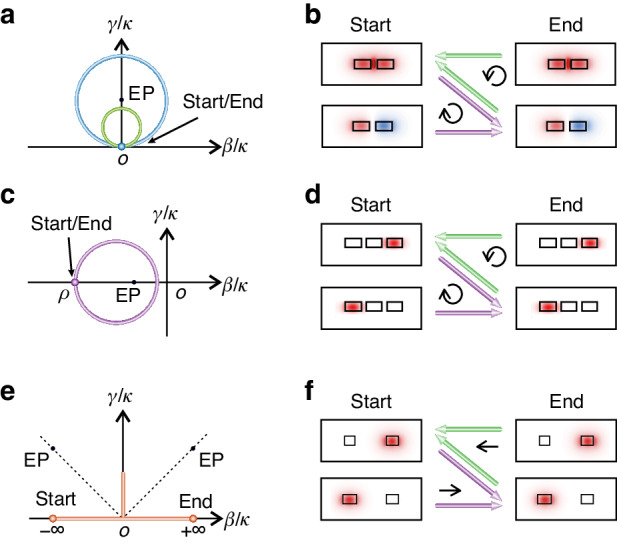
Chiral dynamics with three evolution trajectories. **a,** **b** Closed trajectory in a PT-symmetric system, with evolution trajectory encircling EPs (blue circle) and EP-excluding evolution trajectory (green circle). **c,** **d** Closed trajectory in an anti-PT-symmetric system. **e,** **f** Open trajectory without winding around EPs, and asymmetric mode conversion of asymptotic modes. Two EPs lie at (- ∞, + ∞) and (+ ∞, + ∞), and are not on the evolution trajectory. The circles in (**a**, **c** and **e**) represent the starting and ending points in the evolution trajectories. The purple and green arrows represent clockwise and counterclockwise directions in (**b** and **d**), respectively, and represent the forward and backward directions in (**f**), respectively

Figures 1a–d show the conventional closed evolution trajectories winding around EPs in PT-symmetric and anti-PT-symmetric systems, respectively, where the starting/ending points are the same. The Hamiltonian winding around an EP of a two-level PT-symmetric system composed of two coupled entities has the form^40^

1 $${H}_{{\rm{PT}}}=\left[\begin{array}{cc}\beta /\kappa +i\gamma /\kappa & 1\\ 1 & -\beta /\kappa -i\gamma /\kappa \end{array}\right]$$

where *β*, *γ* and *κ* represent the degree of detuning, loss rate and coupling strength of the system, respectively. The starting/ending points lie at $\beta /\kappa =\gamma /\kappa =0$, with the associated symmetry-preserving modes ${\left[1,\pm 1\right]}^{{\rm{T}}}$, corresponding to the even and odd modes, respectively, for double-coupled waveguides (DCW)^13,15,20,36^. For the two-level anti-PT-symmetric system composed of two coupled entities (Fig. 1c), the Hamiltonian has the form^16^

2 $${H}_{{\rm{APT}}}=\left[\begin{array}{cc}-\beta /\kappa +i\gamma /\kappa & i\\ i & \beta /\kappa -i\gamma /\kappa \end{array}\right]$$

where the starting/ending points lie at $\rho =\beta /\kappa < -1$ and $\gamma /\kappa =0$, with associated symmetry-broken modes ${\left[i,\rho \pm \sqrt{{\rho }^{2}-1}\right]}^{{\rm{T}}}$. This anti-PT-symmetric system can be realized using three-coupled waveguides. When *ρ* approaches -∞, the associated waveguide modes are localized in one waveguide. In both encircling strategies the chiral transmission efficiency is low due to path-dependent losses. It should be noted that, Eq. (1) and Eq. (2) can only satisfy PT symmetry and anti-PT symmetry at *β* = 0 and *γ* = 0, respectively. Here, we have termed the two systems described by Eq. (1) and Eq. (2) as PT symmetry and anti-PT symmetry, respectively, which is consistent with all the previous EP-encircling studies^13,15–18,20,21,36–38^.

Figure 1e presents the proposed open evolution trajectory, in which the starting/ending points are located at two opposite infinite points (± ∞, 0). At these two points, both eigenmodes asymptotically approach ${\left[\mathrm{1,0}\right]}^{{\rm{T}}}$ and ${\left[\mathrm{0,1}\right]}^{{\rm{T}}}$, i.e., each eigenmode is localized in a single waveguide only (Fig. 1f), which allows us to input the same modes at the two terminal points. The crucial NAJ process for inducing the chiral response means the dominant eigenstate of the system is transferred to another eigenstate during the parameter evolution process^15^. Here, it is realized by introducing loss at $\beta /\kappa =0$, and high transmission efficiency can be realized through the judicious control of coupling loss to one of the eigenmodes only. It should be emphasized that, the evolution trajectories encircling EPs in Fig. 1a, c are topologically non-trivial, corresponding to a π Berry phase, while those trajectories without encircling EPs in Figs. 1a and 1e are topologically trivial, associated with a zero Berry phase. The Berry phase governed by topology is invalid in the evolution process described in Fig. 1, due to the occurrence of NAJ. Compared with the evolution trajectories encircling EPs in Figs. 1a and 1c, the EP-excluding evolution trajectory for chiral response (Fig. 1a) requires that the system Hamiltonian evolves sufficiently slowly to ensure the occurrence of NAJ^34^.

**Fig. 2:**
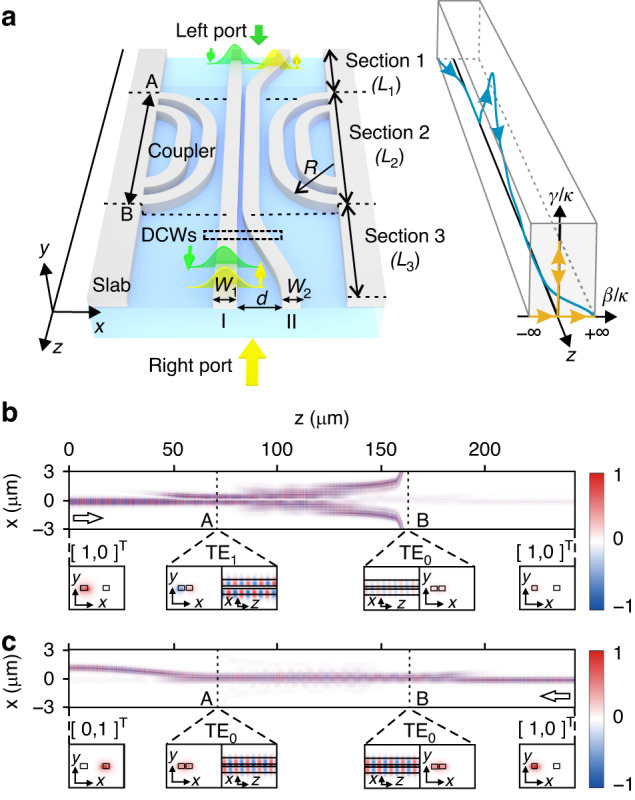
Double coupled waveguides (DCWs) for demonstrating chiral transmission of asymptotic modes. **a** DCWs and adiabatic couplers. *W*_1,2_ is the width of the first and second waveguide, and *d* is the gap distance between them. The DCWs are marked by the dashed rectangle. Ports Ι and ΙΙ correspond to the first and second waveguides, respectively. The blue line with arrows in the right panel shows the evolution trajectory, where its projection onto (β ⁄ κ, γ ⁄ κ) plane is marked by the yellow line. **b,** **c** Simulated field distribution of *E*_*x*_ when the light is injected into port Ι from the left side (**b**) and from the right side (**c**) at 1550 nm

To demonstrate chiral transmission of asymptotic modes with an open Hamiltonian evolution trajectory, shown in Figs. 1e and 1f, we construct an optical system comprised of DCW (waveguide I and II) divided into three sections with boundaries labeled by A and B as shown in Fig. 2a. Waveguide II is wider than waveguide I in Section 1, and this is reversed in Section 3. In Section 2 between A and B, the two waveguides have the same width, and four additional waveguides are added on both sides to function as an adiabatic coupler, such that one eigenmode ${\left[1,-1\right]}^{{\rm{T}}}$ in DCWs is lossy while another eigenmode ${\left[\mathrm{1,1}\right]}^{{\rm{T}}}$ is lossless. The coupled waveguides are fabricated within a silicon-on-insulator (SOI) wafer with a top silicon layer of 220 nm and a SiO_2_ covered layer of 1 μm. The system is designed such that, regardless of the port that the optical energy is injected into from the left side of the DCWs, the output optical energy always exits from port Ι, i.e., ${\left[\mathrm{1,0}\right]}^{{\rm{T}}}$. On the other hand, the output always exits from port ΙΙ, i.e., ${\left[\mathrm{0,1}\right]}^{{\rm{T}}}$, when the optical energy is injected from the right side of the DCWs. Full-wave simulations using finite-difference time-domain (FDTD) methods are performed to confirm the asymmetric mode switching of asymptotic modes. For an input ${\left[\mathrm{1,0}\right]}^{{\rm{T}}}$ in the forward direction, the dominant eigenmode switches to ${\left[\mathrm{1,1}\right]}^{{\rm{T}}}$ in Section 2 because of the NAJ process, and the final energy mainly exits from port Ι, corresponding to ${\left[\mathrm{1,0}\right]}^{{\rm{T}}}$ (Fig. 2b). On the other hand, in the backward direction, the dominant eigenmode is always the symmetrical mode ${\left[\mathrm{1,1}\right]}^{{\rm{T}}}$ in Section 2, and the final optical energy mainly exits from port ΙΙ, corresponding to ${\left[\mathrm{0,1}\right]}^{{\rm{T}}}$ (Fig. 2c).

To understand the dynamics of this intriguing chiral response, this system is described with the evolution equation as^15^

3 $$\frac{d}{{dz}}\left|\psi \right\rangle ={iH}{\rm{|}}\psi {\rm{\rangle }}$$

where the eigenfunction is written as $\left|\psi \left(z\right)\right\rangle ={\left[{b}_{1}\left(z\right),{b}_{2}\left(z\right)\right]}^{{\rm{T}}}$, ${b}_{\mathrm{1,2}}(z)$ are the amplitudes of the modes in each waveguide of the DCWs, and *z* is the propagation distance. By taking into account the energy exchange between the DCWs and the surroundings, the Hamiltonian *H (z)* can be written as (see Supplementary Note 1 for the detailed derivation)

4 $$H\left(z\right)=\left[\begin{array}{cc}\beta \left(z\right)+i\gamma \left(z\right) & \kappa \left(z\right)-i\gamma \left(z\right)\\ \kappa \left(z\right)-i\gamma \left(z\right) & -\beta \left(z\right)+i\gamma \left(z\right)\end{array}\right].$$

The eigenvalues of the Hamiltonian are ${E}_{\mathrm{1,2}}=i\gamma \pm \sqrt{{\beta }^{2}+{\left(\kappa -i{\gamma }\right)}^{2}}$, and the associated eigenstates are ${X}_{\mathrm{1,2}}={\left[\kappa -i{\gamma },\pm \sqrt{{\beta }^{2}+{\left(\kappa -i{\gamma }\right)}^{2}}-\beta \right]}^{{\rm{T}}}$, indicating that the system has EPs at $\beta =\pm \gamma$ with *κ* = 0. Different from the previous PT-symmetric and anti-PT-symmetric systems^13,15–18,20,21,36–38^, the Hamiltonian described by Eq. (4) is neither PT-symmetric nor anti-PT-symmetric. Provided that *H* remains constant over the distance interval $[{z}_{0},z]$, the final state $|\psi (z)\rangle$ can be obtained by incorporating Eq. (4) into Eq. (3),

5 $$\left|\psi \left(z\right)\right\rangle ={c}_{1}\left({z}_{0}\right){e}^{i{E}_{1}(z-{z}_{0})}{X}_{1}+{c}_{2}\left({z}_{0}\right){e}^{i{E}_{2}(z-{z}_{0})}{X}_{2}$$

where the initial state is $\left|\psi \left({z}_{0}\right)\right\rangle ={c}_{1}\left({z}_{0}\right){X}_{1}+{c}_{2}\left({z}_{0}\right){X}_{2}$ at *z*_0_, with *c*_1,2_ being arbitrary coefficients. Equation (5) indicates that the variations of phase and amplitude come from the real and imaginary parts of the eigenvalues, respectively. It is worth emphasizing here that the Hamiltonian formulation extensively used in previous studies on EP encircling in PT-symmetric systems^13,15,17,18,20,21,36–38^ can be equivalently transformed to Eq. (1), and is not able to describe the proposed non-Hermitian systems.

**Fig. 3:**
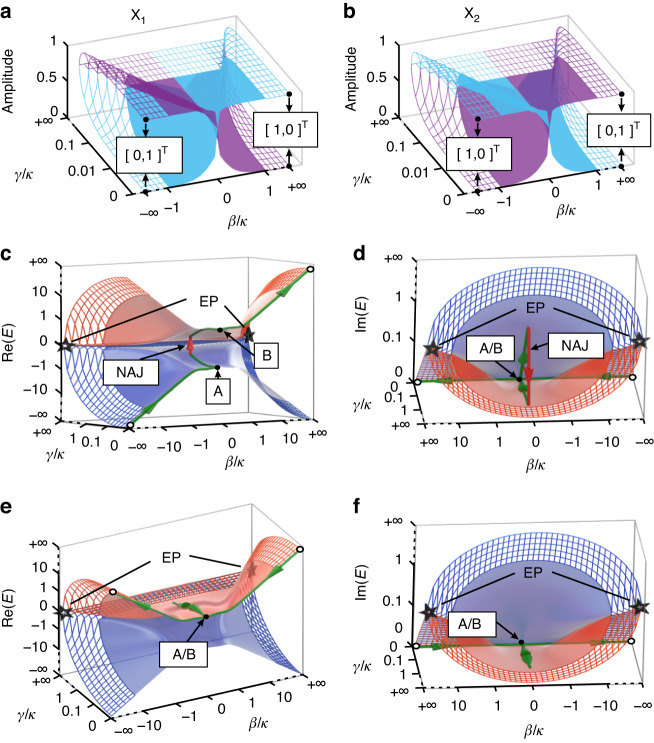
Asymptotic eigenmodes and system states evolving on the Riemann surfaces. **a,** **b** Amplitudes of the normalized eigenmodes *X*_1,2_ of *H* in Eq. (4) in the parameter space (*β ⁄ κ*, *γ ⁄ κ*). The blue and purple sheets represent the amplitudes of the first and second terms of *X*_1,2_. **c,** **d** Forward and **e,** **f**, backward evolution trajectories in the Riemann surfaces formed by the real and the imaginary part of the energy spectra *E* of *H* as the initial state is ${\left[1,0\right]}^{{\rm{T}}}$. The empty circles represent the starting and ending points of the trajectories and the stars represent EPs. The dashed meshes show *β ⁄ κ* and *γ ⁄ κ* extending to infinity. The red and purple sheets denote *E*_1_ and *E*_2_, respectively

For a non-zero *κ*, the amplitudes of the two eigenstates, *X*_1,2_, in the parameter space of *β ⁄ κ* and *γ ⁄ κ* is presented in Fig. 3a, b, indicating that the eigenstate *X*_1_ (*X*_2_) converges asymptotically to ${\left[\mathrm{0,1}\right]}^{{\rm{T}}}$ and ${\left[\mathrm{1,0}\right]}^{{\rm{T}}}$ (${\left[\mathrm{1,0}\right]}^{{\rm{T}}}$ and ${\left[\mathrm{0,1}\right]}^{{\rm{T}}}$) at $\left(\beta /\kappa ,\gamma /\kappa \right)=(-\infty ,0)$ and (+ ∞, 0), respectively. This suggests that two infinite points (± ∞, 0) share the same asymptotic eigenmodes, i.e., ${\left[\mathrm{0,1}\right]}^{{\rm{T}}}$ and ${\left[\mathrm{1,0}\right]}^{{\rm{T}}}$. For all the previously demonstrated evolution strategies with chiral response, a closed trajectory is required to ensure the same initial state for either evolution direction^13,15,20,36^. In contrast, here the same asymptotic eigenmodes located at infinity provide a prerequisite to construct an open evolution trajectory for chiral response.

So far, we have shown that an open evolution trajectory linking two infinities can enable chiral transmission of asymptotic eigenmodes. To enable chiral dynamics, it is crucial to introduce an NAJ process to render the output state different for different evolution directions. In this work, this process is accomplished in the interval of A-B with $\beta /\kappa =0$ and varied *γ ⁄ κ*, where one eigenmode ${\left[1,-1\right]}^{{\rm{T}}}$ is lossy and another one ${\left[\mathrm{1,1}\right]}^{{\rm{T}}}$ is lossless. Figure 3c–f show the dynamic Hamiltonian trajectory between $\left(\beta /\kappa ,\gamma /\kappa \right)=(-\infty ,0)$ and (+ ∞, 0), for forward (Fig. 3c, d) and backward (Fig. 3e, f) directions (corresponding to forward and backward directions of light propagation in DCWs, respectively). For forward direction, the initial state ${\left[\mathrm{1,0}\right]}^{{\rm{T}}}$ at the starting point $\left(\beta /\kappa ,\gamma /\kappa \right)=(-\infty ,0)$ evolves slowly to A on the purple sheet with increased *β ⁄ κ* (Fig. 3c), where the dominant eigenmode is *X*_2_ and the other eigenmode *X*_1_ is infinitesimal. When *γ ⁄ κ* firstly increases and then decreases from A to B, the dominant eigenmode *X*_2_ suffers from high loss and it is completely dissipated. In contrast, *X*_1_ experiences low loss and becomes dominant at B, i.e., an NAJ occurs. The final output mode becomes ${\left[\mathrm{1,0}\right]}^{{\rm{T}}}$ at the terminal point $\left(\beta /\kappa ,\gamma /\kappa \right)=(+\infty ,0)$. The evolution process and final state for backward propagation are quite different, as shown in Fig. 3e, f. The initial state ${\left[\mathrm{1,0}\right]}^{{\rm{T}}}$, associated with *X*_1_, is located on the red sheet of the Riemann surface at $\left(\beta /\kappa ,\gamma /\kappa \right)=(+\infty ,0)$. The system evolves from the start to B as *β ⁄ κ* varies. Next, the system still evolves on the red sheet with firstly increased and then decreased *γ ⁄ κ* between A and B. The mode *X*_1_ is always lossless along this path, because the imaginary part of *E*_1_ is zero. Although a little bit of *X*_2_ is excited, since adiabaticity cannot be strictly fulfilled, its contribution is strongly attenuated between A and B due to the non-zero imaginary part of the eigenvalue of *X*_2_. The Hamiltonian finally reaches the terminal point at $\left(\beta /\kappa ,\gamma /\kappa \right)=(-\infty ,0)$ on the red sheet of the Riemann surface, where the system has a dominant eigenmode ${X}_{1}={\left[\mathrm{0,1}\right]}^{{\rm{T}}}$. The totally different output states for different evolution directions indicate a chiral response. It should be noted that the output states for forward and backward directions are always ${\left[\mathrm{1,0}\right]}^{{\rm{T}}}$ and ${\left[\mathrm{0,1}\right]}^{{\rm{T}}}$, respectively, regardless of the input states. The system state evolution process with ${\left[\mathrm{0,1}\right]}^{{\rm{T}}}$ input can be found in Supplementary Note 2. The open evolution trajectory is also open in the trajectory described by *β*, *γ* and *κ* (see Supplementary Note 3).

The above qualitative analysis can be verified by a quantitative analysis based on a 2 × 2 transmission matrix^41^ (composed of all the four transmission efficiencies for a specific input). The transmission matrices for light traveling from left to right and backward are

6 $$T=\left[\begin{array}{cc}{T}_{11} & {T}_{12}\\ {T}_{21} & {T}_{22}\end{array}\right],{T}^{{\prime} }=\left[\begin{array}{cc}{T}_{11}^{{\prime} } & {T}_{12}^{{\prime} }\\ {T}_{21}^{{\prime} } & {T}_{22}^{{\prime} }\end{array}\right]$$

respectively, where *T*_*mn*_ (${T}_{{mn}}^{{\prime} }$) represents the transmission efficiency of the output port *m* at the right (left) of the device when light inputs from the port *n* at the left (right). Because of reciprocity, ${T}_{{mn}}={T}_{{nm}}^{{\prime} }$ must be satisfied. By assuming that the energy transfer coefficient (*K*) from one eigenmode to another eigenmode is small in Sections 1 and 3 (adiabaticity condition), ${T}_{11}={T}_{22}=K$. In Section 2, since the eigenmode ${\left[1,-1\right]}^{{\rm{T}}}$ has a loss of Γ and the eigenmode ${\left[\mathrm{1,1}\right]}^{{\rm{T}}}$ is lossless, ${T}_{12}\approx 0$ and ${T}_{21}\approx \max (\Gamma ,2K)$ (in unit of dB), where $\Gamma =10\mathrm{lg}\{\exp [-{\int }_{{z}_{A}}^{{z}_{B}}4\gamma (z){dz}]\}$ is the accumulated loss for the ${\left[1,-1\right]}^{{\rm{T}}}$. The values of each element of *T* (*T*’) can be obtained and they are (see Supplementary Note 4 for the theoretical derivation)

7 $$T=\left[\begin{array}{cc}K & 0\\ \max (\Gamma ,2K) & K\end{array}\right].$$

The condition that an NAJ occurs and the output is mainly localized at port Ι at the right side (${\left[\mathrm{1,0}\right]}^{{\rm{T}}}$) for two inputs (${\left[\mathrm{1,0}\right]}^{{\rm{T}}}$ and ${\left[\mathrm{0,1}\right]}^{{\rm{T}}}$) from left side is ${T}_{11} > {T}_{21}$ and ${T}_{12} > {T}_{22}$, i.e., $\Gamma < K$. Under the same condition, the output is mainly localized at port ΙΙ at left side (${\left[\mathrm{0,1}\right]}^{{\rm{T}}}$) for two inputs (${\left[\mathrm{1,0}\right]}^{{\rm{T}}}$ and ${\left[\mathrm{0,1}\right]}^{{\rm{T}}}$) from right side. The different output modes demonstrate chiral transmission. If lower loss is considered, i.e., $\Gamma > K$, the output is mainly localized at port ΙΙ (Ι) at one side for the input at port Ι (ΙΙ) at another side, regardless of the direction. Therefore, $\Gamma =K$ is a transition point, above which ($\Gamma \gg K$, lower loss) the evolution of the system follows the adiabatic and symmetric evolution, and below this value ($\Gamma < K$, larger loss) the NAJ process will dominate so that the evolution is asymmetric.

To validate chiral transmission of asymptotic modes by a coupled-waveguide system, as shown in Fig. 2a, the Hamiltonian parameters (*β*, *γ* and *κ*) are theoretically retrieved based on coupled mode theory^42^ and the Beer-Lambert-Bouguer law^43^. The detailed derivation and relationship between the Hamiltonian and geometrical parameters can be found in Supplementary Note 5. We start by converting the selected Hamiltonian parameters at 1550 nm into geometrical parameters of the waveguides (see detailed structure, Hamiltonian parameters and evolution trajectory described by *β / κ* and *γ / κ* in Supplementary Note 6). Subsequently, the Hamiltonian parameters at 1500 and 1600 nm are calculated based on the geometrical parameters. Both ports corresponding to infinite points and the adiabatic coupler introduced to exert coupling loss selectively onto the odd eigenmode ${\left[1,-1\right]}^{{\rm{T}}}$, are illustrated in Supplementary Note 6. The evolution process ensures the occurrence of NAJ in A-B interval, but it also avoids the insurgence of loss except for the NAJ process, as opposed to conventional loops encircling EPs that suffer from path-dependent losses^13,15,16,20,36^.The dynamic process further confirms the chiral response, i.e., different modes output for opposite evolution handedness with the same modes input, as well as high-efficiency chiral transmission between ${\left[\mathrm{1,0}\right]}^{{\rm{T}}}$ and ${\left[\mathrm{0,1}\right]}^{{\rm{T}}}$ (see Supplementary Note 7 for the dynamics of evolution trajectories and the definition of chiral transmission efficiency).

**Fig. 4:**
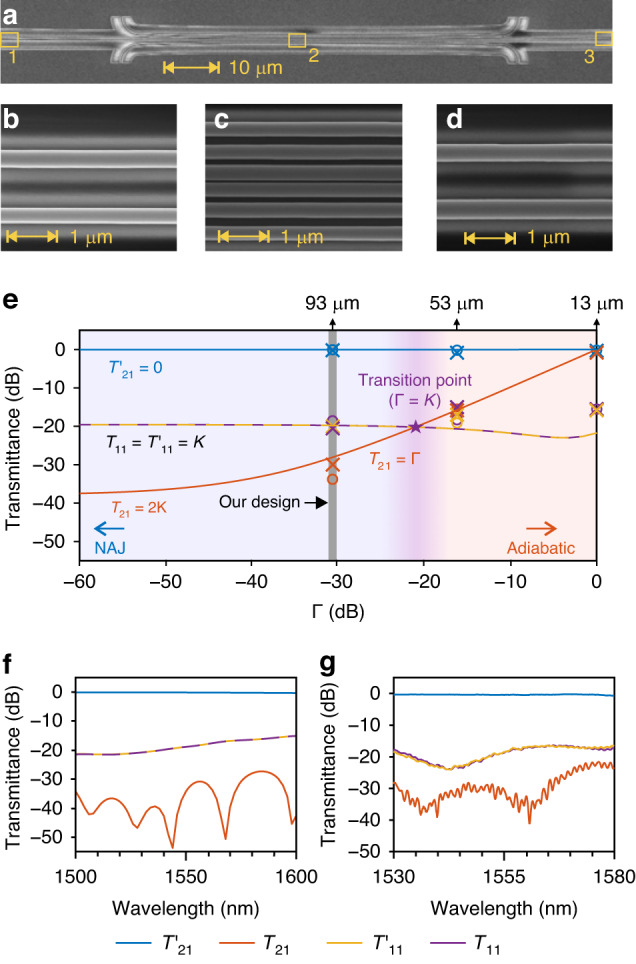
Experimental demonstration of chiral transmission. **a** SEM image of the device. **b**–**d** Zoom-in SEM images bounded by the rectangles are marked with yellow lines in (**a**), numbered by 1, 2 and 3, respectively. **e** Theoretical, simulated and experimental transmittance versus Γ at 1550 nm. The symbols ○ and × represent the simulated and experimental transmittance with different lengths *L*_2_ = 13, 53, 93 μm. **f,** **g** Simulated (**f**) and experimental (**g**) transmittance spectra for the output ports over the wavelength range of 1500–1600 nm and 1530–1580 nm, respectively, with *L*_2_ = 93 μm

The sample was fabricated with a combination of electron-beam lithography and inductively coupled plasma etching, followed by plasma enhanced chemical vapor deposition to cover the entire sample with a 1-μm-thick layer of SiO_2_. Scanning electron microscope (SEM) images for the DCWs in one of the fabricated samples are shown in Fig. 4a–d, where the zoomed-in images in Fig. 4b–d illustrate the regions bounded by the rectangles marked with yellow lines in Fig. 4a, labeled as 1, 2 and 3, respectively. Grating couplers were placed on both sides of DCWs for transmission measurement. The control device without the DCWs is introduced to evaluate the transmittance at different ports by comparing the loss difference between the fabricated sample consisting of DCWs and the control device without DCWs (see more details on fabrication and measurement in Supplementary Note 8).

The transmission described in Eq. (7) can be retrieved by numerically integrating Eq. (3) with the Hamiltonian parameters (*β*, *γ* and *κ*). The presented values in Fig. 4e are well consistent with simulations and experiments, as the accumulated loss Γ is varied by changing the coupler lengths between A and B. As illustrated in Fig. 4e, the transmission is symmetrical when Γ = 0 dB, corresponding to the length of Section 2 is *L*_2_ = 13 μm (almost the length of 2*R* in Fig. 2a). When Γ is reduced to −17 dB (*L*_2_ = 53 μm), close to the transition point (*K* = −20 dB), the output light energy at ports Ι and ΙΙ at right side is almost equal for input at port Ι. When Γ is further reduced to be approximately −30 dB (*L*_2_ = 93 μm) so that the NAJ occurs, the transmission process is asymmetrical since the output light energy is mainly localized at port Ι at right side. If Γ is further reduced, it is required to exponentially increase the length of the adiabatic coupler. In this situation, only *T*_21_ can be slightly reduced, while all other transmission efficiencies, ${T}_{21}^{{\prime} }$, ${T}_{11}$, ${T}_{11}^{{\prime} }$, are almost kept unchanged. In a word, the device performance hardly improves by further increasing the length of the adiabatic coupler. Consequently, it is unnecessary to significantly increase the length of the adiabatic coupler, from the viewpoint of device size and fabrication. Both simulated and experimental results of asymmetric transmission in Fig. 4f and g demonstrate high transmission efficiency over a wide telecommunication wavelength region for the asymptotic mode of ${\left[\mathrm{0,1}\right]}^{{\rm{T}}}$ (the optical field is localized in the second waveguide) on the left side, when the asymptotic mode of ${\left[\mathrm{1,0}\right]}^{{\rm{T}}}$ (the optical field is localized in the first waveguide) is excited on the right side. The chiral transmission efficiency, ${T}_{21}^{{\prime} }$, is close to 100% within the wavelength range of interest. It should be noted that the output mode is still ${\left[\mathrm{1,0}\right]}^{{\rm{T}}}$ (${\left[\mathrm{0,1}\right]}^{{\rm{T}}}$), when ${\left[\mathrm{0,1}\right]}^{{\rm{T}}}$ is injected from left (right) side (Supplementary Note 9).

It is worth comparing the introduced open trajectory with the conventional closed trajectories that wind around EPs for chiral transmission. Most of previous studies involving PT-symmetric systems rely on path-dependent losses for encircling EPs, resulting in low chiral transmission between symmetrical and anti-symmetrical modes^13,15,20,36^. The evolution strategies using Hamiltonian hopping and fast encirclement of EPs in PT-symmetric systems can boost this chiral transmission efficiency to near unity^37,38^. However, realizing chiral transmission of symmetry-broken modes, which is more accessible in practical photonic integrated circuits since each eigenmode is localized in a single waveguide, has been proven challenging in PT-symmetric systems. While promising steps towards chiral transmission of symmetry-broken modes have been taken by anti-PT-symmetric systems^16^, the chiral transmission efficiency is ultra-low (around 4% in experiment) because it is also limited by path-dependent losses in EP encircling. In contrast to the previously studied PT-symmetric and anti-PT-symmetric systems, the proposed open trajectory without EP encircling is conducted in a non-Hermitian system that is neither PT-symmetric nor anti-PT-symmetric. It can not only enable the chiral transmission of the asymptotic mode, i.e., the light field is localized in a single waveguide, but also achieve high-efficiency chiral transmission by selectively exerting coupling loss to one of the eigenmodes during the evolution. Consistent with the chiral transmission study based on Hamiltonian hopping and fast encirclement of EPs, we have also used the silicon platform to experimentally demonstrate the chiral transmission. The difficulty and complexity of the silicon waveguides arising during the fabrication process mainly come from the number of EBL process steps and precise alignment. The chiral converters with the proposed open trajectory and Hamiltonian hopping share the same fabrication difficulty, since only one-step EBL is required for them. In contrast, experimental demonstration of chiral transmission based on fast encirclement of EPs is more challenging, since three-step EBL and precise alignment between the chromium layer and silicon waveguides are required.

## Discussion

In conclusion, we have demonstrated a counterintuitive chiral dynamic system by considering a Hamiltonian evolving along an open trajectory without EP encircling. The NAJ process that is crucial for chiral dynamics is realized by exerting loss on the asymmetric mode only during evolution, which ensures the occurrence of NAJ but does not introduce any loss to the system except for NAJ process. We have experimentally demonstrated high-efficiency chiral transmission of asymptotic modes that the optical field is localized at one waveguide only among double-coupled waveguides. The chiral transformation combined with nonlinear systems opens opportunities for applications in the context of optical nonreciprocity^44^. The chiral transmission of asymptotic modes is expected to develop single-port output laser on a chip with tailored gain and loss^45^. The strategy of selectively introducing gain or loss to specific modes might be beneficial for locking spatial modes of output lasers in coupled waveguides laser arrays, which could be developed for laser emission from a single-port waveguide that is advantageous to enhance on-chip output power and reduce laser threshold^46^. The chiral-transmission effect might be introduced in quantum walk systems for the generation of quantum entangled states that are insensitive to input states, suggesting promising applications in quantum information processing and quantum communication^47^. Our results offer a new approach to study chiral dynamics in non-Hermitian systems, and open new avenues for the development of practical asymmetric-transmission devices and applications.

## Materials and methods

### Fabrications

The fabrication of the devices is combined with three-step electron-beam lithography (EBL), inductively coupled plasma (ICP) etching, electron-beam evaporation (EBE), and plasma-enhanced chemical vapor deposition (PECVD). The first-step EBL and EBE is aimed to form the Au marks on an SOI wafer for alignment. The second-step EBL and ICP is used to define the grating couplers. The DCW is fabricated by the third-step EBL and ICP. Finally, PECVD is applied to deposit a 1-μm-thick SiO_2_ cladding layer to cover the entire device.

### Measurements

An amplified spontaneous emission (ASE) source (OVLINK ASE-CL-20-B) provides the near-infrared light, and its polarization is adjusted by a polarizing beam splitter (PBS) and a polarization controller. The grating coupler is used to couple the light from the fiber into TE_0_ mode or decouple the TE_0_ mode out of the silicon waveguide back into the fiber. The decoupled light will be collected by the optical power meter (PMSII-A) and a spectrometer (YOKOGAWA AQ6370C). See more details on the measurements in Supplementary Note 8.

## Availability of data and material

Structural parameters, simulated and experimental data have been provided within the main text and Supplementary Material of this paper. All the other data that support the findings of this study are available from the corresponding author upon reasonable request. The code that supports the plots within this paper is available from the corresponding authors upon reasonable request.

### Supplementary information

Supplementary material for Chiral transmission by an open evolution trajectory in a non-Hermitian system
