# Supplementary material for: Chiral transmission by an open evolution trajectory in a non-Hermitian system
Source: Light Sci Appl. 2024 Mar 5;13:65. doi: 10.1038/s41377-024-01409-1 (PMC10912664; doi:10.1038/s41377-024-01409-1)
Supplement: Supplementary file 1 — Supplementary material for Chiral transmission by an open evolution trajectory in a non-Hermitian system [file 41377_2024_1409_MOESM1_ESM.pdf]

Supplementary material for

Chiral transmission by an open evolution trajectory in a non-Hermitian system

Xiaoqian Shu[1, 6,†], Qi Zhong[2,†], Kai Hong[1], Oubo You[3], Jian Wang[1], Guangwei Hu[4], Andrea Alù[5], Shuang Zhang[3], Demetrios N. Christodoulides[2] and Lin Chen[1,7,*]

[1]Wuhan National Laboratory for Optoelectronics and School of Optical and Electronic Information, Huazhong University of Science and Technology, Wuhan 430074, China

[2]CREOL, College of Optics and Photonics, University of Central Florida, Orlando, Florida 32816, USA

[3]Department of Physics, The University of Hong Kong, Hong Kong, China

[4]School of Electrical and Electronic Engineering, Nanyang Technological University, 50 Nanyang Avenue, Singapore, 639798 Singapore

[5]Photonics Initiative, Advanced Science Research Center, City University of New York, New York, NY 10031, USA

[6]Zhejiang Lab, Hangzhou 311121, China

[7]Shenzhen Huazhong University of Science and Technology Research Institute, Shenzhen 518063, China

Email: chen.lin@mail.hust.edu.cn.

* Corresponding author.

† These authors contributed equally to this work.

Supplementary Note 1: Derivation of evolution equation

In sections 1 and 3, $0 < z < z_A$ or $z_B < z < z_{end}$, the coupled mode equation can be written as

$$\begin{aligned} \frac{da_1}{dz} &= i\beta_1 a_1 + i\kappa a_2 \\ \frac{da_2}{dz} &= i\kappa a_1 + i\beta_2 a_2 \end{aligned}, \tag{S1}$$

where a_1 and a_2 are the amplitude of the system state, β_1 and β_2 are the propagation constants, and κ is the coupling coefficient. Eq. S1 can be rewritten as

$$\frac{d}{dz}\begin{bmatrix} a_1 \\ a_2 \end{bmatrix} = i\begin{bmatrix} \beta_1 & \kappa \\ \kappa & \beta_2 \end{bmatrix}\begin{bmatrix} a_1 \\ a_2 \end{bmatrix}, \tag{S2}$$

In section 2, $z_A < z < z_B$, the eigenstates of two middle waveguides with same width are $X_1 = [1,1]^{\mathrm{T}}$ and $X_2 = [1,-1]^{\mathrm{T}}$. Owning to lossless X_1 and lossy X_2, the corresponding eigenvalues are assumed to be $E_1(z) = \lambda_1(z)$ and $E_2(z) = \lambda_2(z) + i\lambda'(z)$ ($\lambda_{1,2}$ and λ' are real numbers). Consequently, the Hamiltonian satisfying these eigenstates and eigenvalues simultaneously is

$$H'(z) = \begin{bmatrix} \frac{\lambda_1 + \lambda_2}{2} + i\frac{\lambda'}{2} & \frac{\lambda_1 - \lambda_2}{2} - i\frac{\lambda'}{2} \\ \frac{\lambda_1 - \lambda_2}{2} - i\frac{\lambda'}{2} & \frac{\lambda_1 + \lambda_2}{2} + i\frac{\lambda'}{2} \end{bmatrix}, \tag{S3}$$

Defining $\beta'(z) = \frac{\lambda_1(z) + \lambda_2(z)}{2}$, $\kappa'(z) = \frac{\lambda_1(z) - \lambda_2(z)}{2}$, and $\gamma'(z) = \frac{\lambda'(z)}{2}$, Eq. S3 can be rewritten as

$$H'(z) = \begin{bmatrix} \beta' + i\gamma' & \kappa' - i\gamma' \\ \kappa' - i\gamma' & \beta' + i\gamma' \end{bmatrix}, \tag{S4}$$

The formula of coupling length is $\frac{\pi}{k_0\left(n_{\mathrm{even}} - n_{\mathrm{odd}}\right)}$, with wave number k_0, effective refractive indices, n_{even} and n_{odd} for even and odd modes, associated with

$\kappa' - i\gamma' = \frac{1}{2} k_0 \left(n_{\text{even}} - n_{\text{odd}} \right)$. In principle, we can simultaneously introduce loss to even and odd modes to generate a non-zero value of γ'. In our practical design of the coupled waveguide system, we only exert loss to odd mode by attaching the adiabatic couplers.

Comparing Eq. S2 and S4, the evolution equation for the entire system can be written as

$$\frac{d}{dz}\begin{bmatrix} a_1 \\ a_2 \end{bmatrix} = \begin{bmatrix} \beta_1 + i\gamma & \kappa - i\gamma \\ \kappa - i\gamma & \beta_2 + i\gamma \end{bmatrix} \begin{bmatrix} a_1 \\ a_2 \end{bmatrix}, \tag{S5}$$

with the definition of $b_{1,2} = a_{1,2} \exp\left[-i\left(\frac{\beta_1 + \beta_2}{2} \right) z \right]$, Eq. S5 can be transformed to

$$\frac{d}{dz}\begin{bmatrix} b_1 \\ b_2 \end{bmatrix} = \begin{bmatrix} \beta + i\gamma & \kappa - i\gamma \\ \kappa - i\gamma & -\beta + i\gamma \end{bmatrix} \begin{bmatrix} b_1 \\ b_2 \end{bmatrix}, \tag{S6}$$

with $\beta = \frac{\beta_1 - \beta_2}{2}$. When $0 < z < z_A$ or $z_B < z < z_{end}$, $\gamma = 0$. When $z_A < z < z_B$, $\beta = 0$.

Supplementary Note 2: Forward and Backward evolution process with initial state $[0,1]^{\mathrm{T}}$

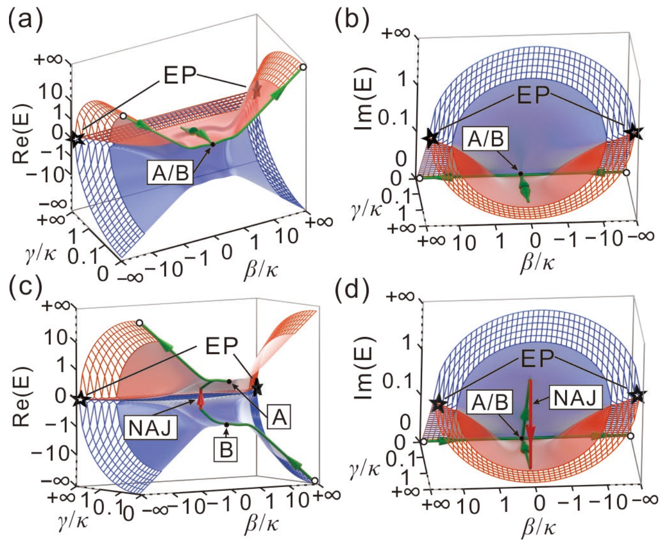

FIG. S1 | System states evolving on the Riemann surfaces. (a, b) Forward and (c, d) backward paths in the Riemann surfaces formed by the real part $\mathrm{Re}(E)$ and the imaginary part $\mathrm{Im}(E)$ of the energy spectra of H as the initial state is $[0,1]^{\mathrm{T}}$.

Figure S1 shows the dynamic evolution paths of the Hamiltonian for forward (Figs. S1(a) and S1(b)) and backward evolution [Figs. S1(c) and S1(d)]. As for forward evolution [Figs. S1(a) and S1(b)], the initial state $[0,1]^T$ is located on the red sheet of the Riemann surface at the starting point $(\beta/\kappa, \gamma/\kappa) = (-\infty, 0)$. X_1 is always lossless and dominant as the imaginary part of E_1 is always zero. In contrast, X_2 is infinitesimal and attenuated when evolving from A to B. The Hamiltonian finally exits at $(\beta/\kappa, \gamma/\kappa) = (+\infty, 0)$ with the output state dominated by $X_1 = [1,0]^T$ on the red sheet of the Riemann surface. For backward evolution, the initial state $[0,1]^T$ is located on the blue sheet of the Riemann surface at the starting point $(\beta/\kappa, \gamma/\kappa) = (+\infty, 0)$. [Figs. S1(c) and S1(d)]. When the initial state evolves slowly to B with X_2 dominant,

X_1 is triggered and infinitesimal as adiabatic evolution condition is not strictly fulfilled. X_2 incurs complete dissipation during the interval between A and B as γ/κ firstly increases to a maximum value and then decreases to 0. Therefore, the lossless eigenstate X_1 dominates at A, i.e., NAJ occurs. The final state returns to $[0,1]^{\mathrm{T}}$ at $(\beta/\kappa, \gamma/\kappa) = (-\infty, 0)$.

Supplementary Note 3: The evolution trajectory in 3D parameter space

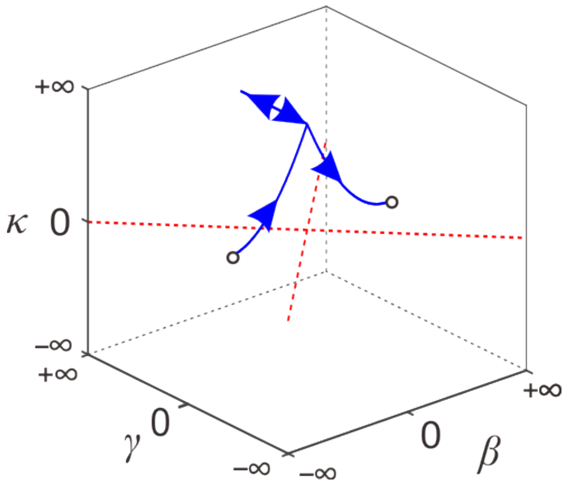

FIG. S2 | The evolution trajectory in 3D parameter space described by (β, γ, κ). The blue lines indicate the evolutionary trajectory and the red lines indicate the EP lines.

Supplementary Note 4: Derivation of transmission matrices

First, we derive the expression for T_{11}. The input at port I at left side corresponds to the supermode $[1,0]^T$. Because of the imperfect adiabaticity condition in section 1, most of the power will stay on the supermode $[1,-1]^T$ at position A (denoted as τ_1^2, and $\tau_1^2 \sim 1$) because of the adiabatic evolution, while a small percent of power (denoted as k_1^2, $k_1^2 << 1$, and $\tau_1^2 + k_1^2 = 1$ because of energy conservation) will be coupled to another supermode $[1,1]^T$. When light travels in section 2, the power of $[1,-1]^T$ component will decay to $\tau_1^2 \cdot \tilde{\gamma}$ at position B, in which $\tilde{\gamma} = \exp\left[-\int_{z_A}^{z_B} 4\gamma(z)\,dz\right]$ is the loss in section 2. On the other hand, the $[1,1]^T$ component would not experience any loss and it is still k_1^2 at position B. In section 3, the power at port I at the right side (corresponding to mode $[1,0]^T$) consists of two parts: one is from the transmission of $[1,1]^T$, i.e., $k_1^2 \cdot 1 \cdot \tau_3^2$; and the second part is the coupling from mode $[1,-1]^T$, i.e., $\tau_1^2 \cdot \tilde{\gamma} \cdot k_3^2$. Here k_3^2 stands for the power transfer between supermodes at section 3, and in our design $k_3^2 \sim k_1^2$. Therefore, we can write the total power at port I at the right side as $T_{11} \approx k_1^2 \cdot 1 \cdot \tau_3^2 + \tau_1^2 \cdot \tilde{\gamma} \cdot k_3^2$. Note that here the summation of power is an approximation under the condition that one term is much larger than another term (an accurate approach should be the summation of field b_i instead of the power $|b_i|^2$, and this can be done by the transfer matrix approach). In the expression of T_{11}, under the condition that $\tilde{\gamma} << 1$, the first term dominates, and therefore $T_{11} \approx k_1^2 \tau_3^2 \approx k_1^2$.

Similarly, the value of other elements of T can be derived [1] and that is:

$$T_{21} \approx k_1^2 \cdot 1 \cdot k_3^2 + \tau_1^2 \cdot \tilde{\gamma} \cdot \tau_3^2 \approx \begin{cases} \tilde{\gamma}, & \tilde{\gamma} >> k_1^2 k_3^2 \\ k_1^2 k_3^2, & k_1^2 k_3^2 >> \tilde{\gamma} \end{cases},$$

$$T_{12} \approx \tau_1^2 \cdot 1 \cdot \tau_3^2 + k_1^2 \cdot \tilde{\gamma} \cdot k_3^2 \approx \tau_1^2 \tau_3^2 \approx 1,$$

$$T_{22} \approx \tau_1^2 \cdot 1 \cdot k_3^2 + k_1^2 \cdot \tilde{\gamma} \cdot \tau_3^2 \approx k_3^2 .$$

By writing $K_{1,3} = 10\lg\left(k_{1,3}^2\right)$, and $\Gamma = 10\lg\left(\tilde{\gamma}\right)$, the transmission matrix T (in units of dB) is

$$T = \begin{bmatrix} T_{11} & T_{12} \\ T_{21} & T_{22} \end{bmatrix} = \begin{bmatrix} K_1 & 0 \\ \max(\Gamma, K_1 + K_3) & K_3 \end{bmatrix}, \tag{S7}$$

In our design, $K_1 \approx K_3 \equiv K$, therefore we simplify Eq. (S7) as

$$T = \begin{bmatrix} T_{11} & T_{12} \\ T_{21} & T_{22} \end{bmatrix} = \begin{bmatrix} K & 0 \\ \max(\Gamma, 2K) & K \end{bmatrix}, \tag{S8}$$

Because of reciprocity, the transmission matrix T' for excitation from right side is

$$T' = \begin{bmatrix} T'_{11} & T'_{12} \\ T'_{21} & T'_{22} \end{bmatrix} = \begin{bmatrix} T_{11} & T_{21} \\ T_{12} & T_{22} \end{bmatrix} = \begin{bmatrix} K & \max(\Gamma, 2K) \\ 0 & K \end{bmatrix}. \tag{S9}$$

Supplementary Note 5: Calculation of Hamiltonian parameters

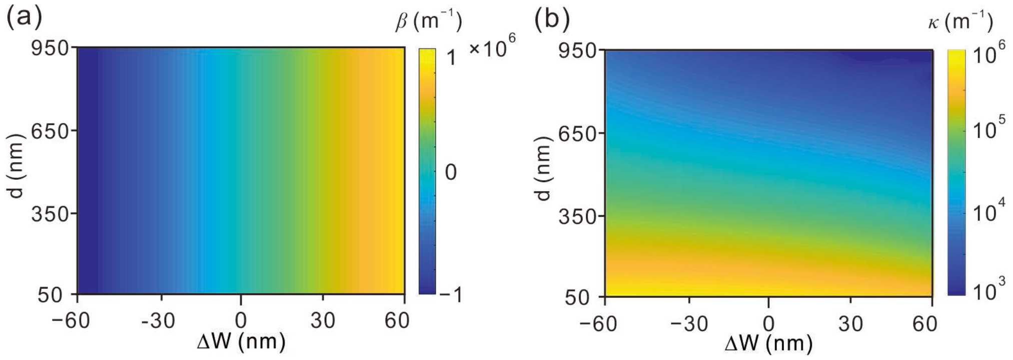

FIG. S3 | Hamiltonian parameters. (a, b) β and κ versus the width difference, $\Delta W = W_1 - W_2$, and the gap distance d of two middle waveguides at 1550 nm.

The dependence of β and κ on ΔW and d is depicted in Fig. S3. β and κ used in the trajectory can be selected accordingly. The calculation of γ is based on Beer-Lambert-Bouguer law [1]. With the assumption of the complex refractive index of the optical absorbing medium, $\tilde{n} = n' + in''$, the optical intensity in the medium is

$$I = I_0 \exp\left(-\bar{\alpha} z\right), \tag{S10}$$

where I_0 is the optical intensity at $z = 0$, $\bar{\alpha} = \frac{2n''\omega}{c}$ is the attenuation constant with angular frequency ω and velocity of light in vacuum c, z is propagation distance. As a result, $\bar{\alpha} = 4\gamma$, and γ is accessible according to the distributions of $I(z)$ along z direction in two middle waveguides.

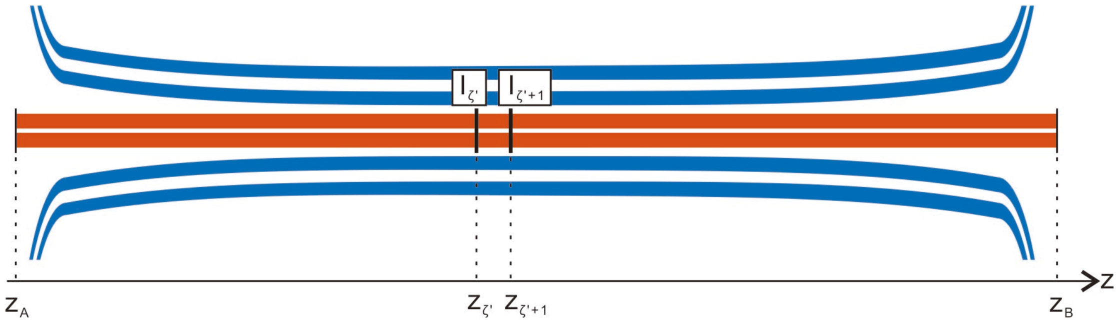

FIG. S4 | Calculation of $\gamma(z)$ by the adiabatic coupler. Two middle waveguides are denoted with red and four side waveguides are denoted with blue.

As shown in Fig. S4, the adiabatic coupler is equally divided into ζ sections. When ζ is big enough, the attenuation constant in each section remains constant. According to Eq. S7, the optical intensity at $z = z_{\zeta'}$ ($\zeta' = 0,1,2,...,\zeta$) is

$$I_{\zeta'+1} = I_{\zeta'} \exp\left[-\bar{\alpha}_{\zeta'}\left(z_{\zeta'+1} - z_{\zeta'}\right)\right], \tag{S11}$$

Hence, inputting $[1,-1]^T$ into the adiabatic coupler at $z = z_A$ in simulation, $\gamma(z)$ can be obtained from $\bar{\alpha}(z)$. According to simulation results, the maximum value of γ reaches around 4×10^4 m^{-1}.

Supplementary Note 6: Structural and Hamiltonian parameters

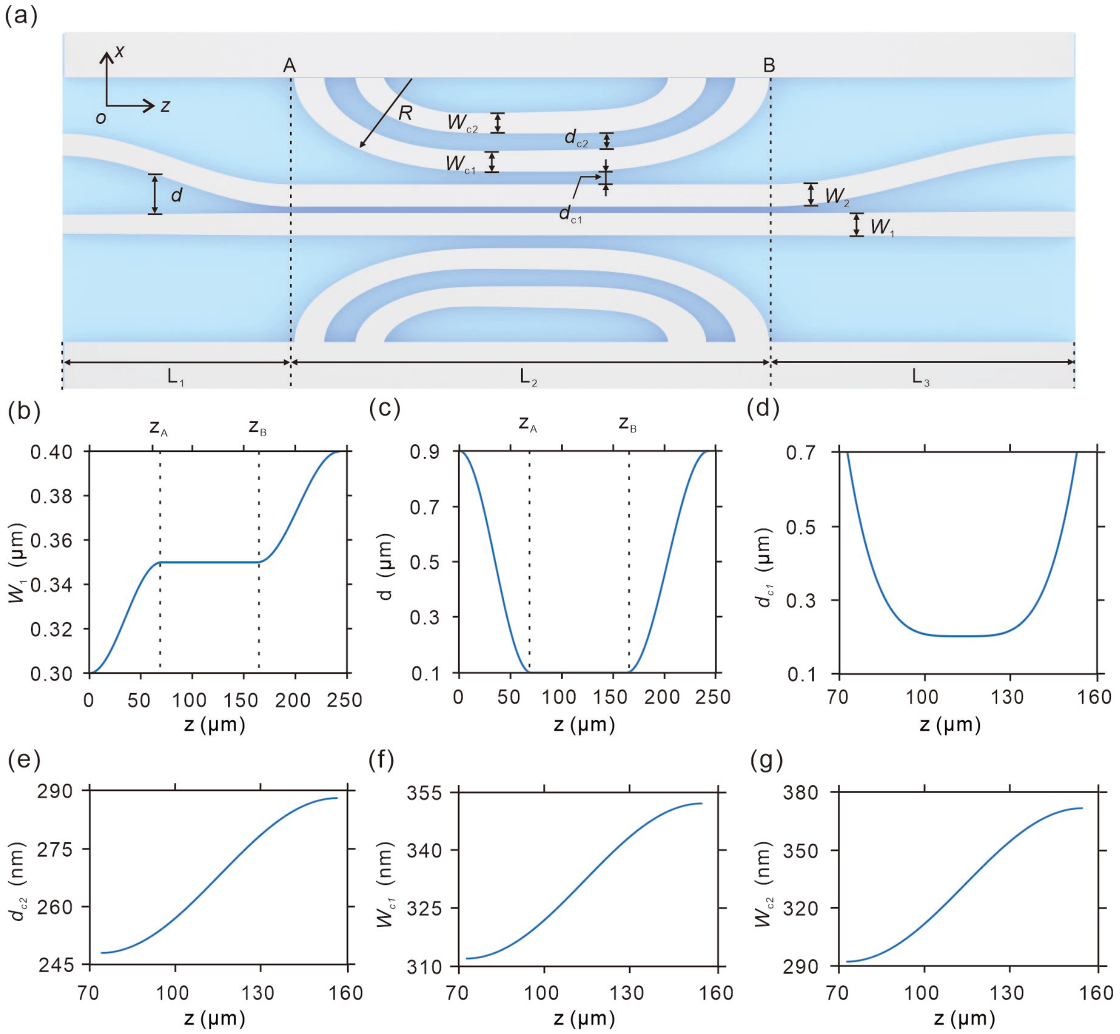

FIG. S5 | Structural parameters of the coupled waveguides. (a) The top view of coupled silicon waveguides. (b-g) The width of the first middle waveguide, W_1, the gap distances of two middle waveguides and two side waveguides, d, d_{c1} and d_{c2}, the widths of two side waveguides, W_{c1} and W_{c2}, versus *z*.

Figure S5(a) schematically shows the structural configuration of double-coupled waveguides (DCWs). The dependence of W_1, d, d_{c1}, d_{c2}, W_{c1} and W_{c2} on *z* is shown in Figs. S5(b)-S5(g), respectively. The other geometrical parameters are $W_2 = 0.35$ μm, $R = 5.9$ μm, $L_1 = 70$ μm, $L_2 = 93$ μm and $L_3 = 80$ μm. Four side waveguides are longitudinally symmetric.

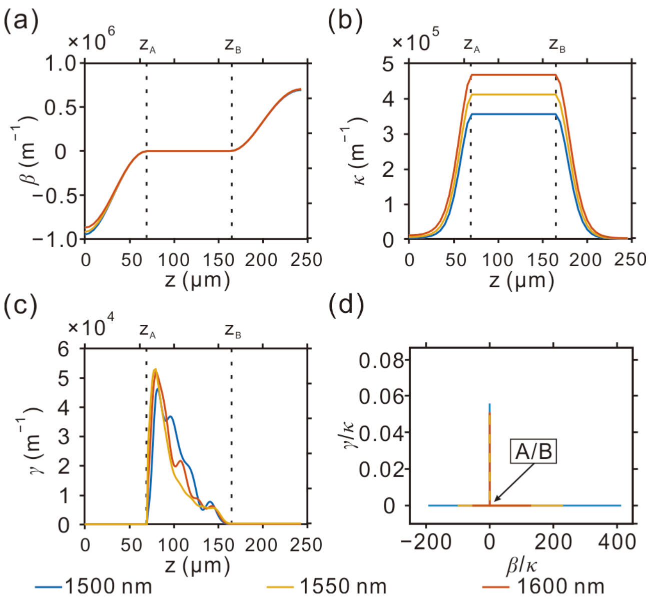

FIG. S6 | Hamiltonian parameters used in the path. (a-c) Dependence of β (a), κ (b), and γ (c) on the spatial coordinate z at 1500, 1550, and 1600 nm. (d) Evolution path at 1500, 1550, and 1600 nm. A and B are the points of division in the evolution.

The two coupled waveguides have a larger gap separation at the terminal points, associated with a smaller κ. Because of the larger width of Waveguide II than that of Waveguide I in the first section, and is reversed in the third section, Section 1 and Section 3 associate with a negative and positive β, respectively [Fig. S6(a)]. Consequently, the cross sections for the left and right sides correspond to the infinite points $(-\infty,0)$ and $(+\infty,0)$, respectively. In the interval between $(-\infty,0)$ and A, or B and $(+\infty,0)$, the gap separation is varied, associated with a continuously-varied κ [Fig. S6(b)]. In this case, the DCWs in the first and third sections do not exchange energy with the surroundings, and the Hamiltonian evolves from $(-\infty,0)$ to point A $(0,0)$ and from point B $(0,0)$ to $(+\infty,0)$, for which the system does not suffer from any loss [Fig. S6(c)]. For the intermediate process from A to B, the adiabatic coupler, consisting of the DCWs and four bending waveguides on the side, is used to introduce coupling loss to the odd eigenmode, $[1,-1]^T$, but does not exert any influence on the even eigenmode, $[1,1]^T$. The evolution process ensures the occurrence of NAJ in A-B interval, but also avoids the insurgence of loss except for the NAJ process [Fig. S6(d)].

Supplementary Note 7: Dynamics of evolution trajectories

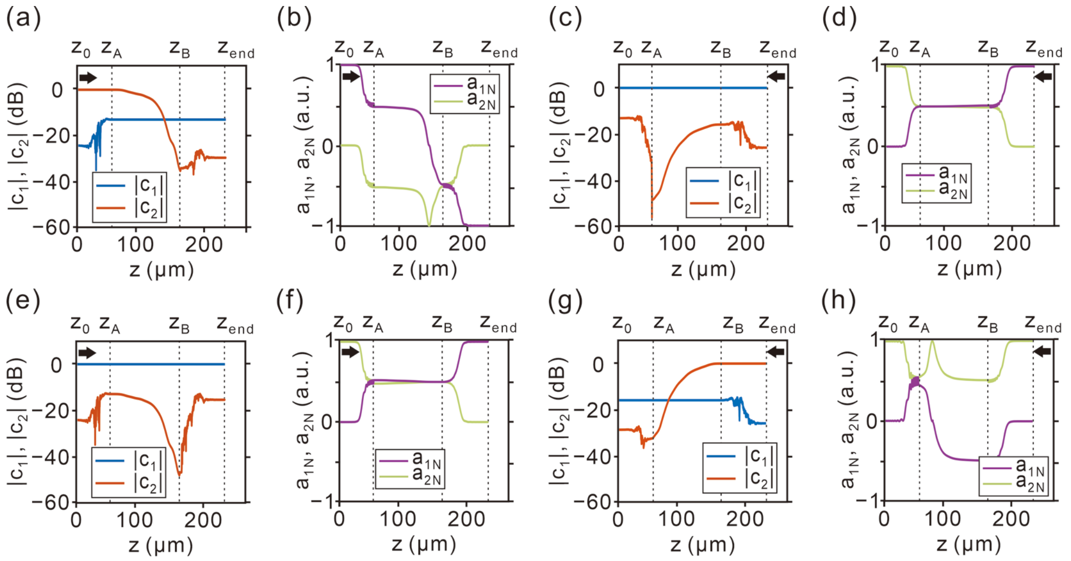

FIG. S7 | Dynamics of the unclosed trajectories. (a-d) The coefficients, $|c_1|$, $|c_2|$, and normalized states, a_{1N}, a_{2N} versus the propagation distance, z, in (a, b) forward and (c, d) backward directions, when the initial state is $[1,0]^T$. (e-h) The coefficients, $|c_1|$, $|c_2|$, and normalized states, a_{1N}, a_{2N} versus z in (e, f) forward and (g, h) backward directions, when the initial state is $[0,1]^T$. The normalized states are defined as $a_{1N} = \mathrm{sign}(a_1) \times |a_1|^2 / (|a_1|^2 + |a_2|^2)$ and $a_{2N} = \mathrm{sign}(a_2) \times |a_2|^2 / (|a_1|^2 + |a_2|^2)$, where $\mathrm{sign}(a_1)$ and $\mathrm{sign}(a_2)$ are used to reflect the phase difference at z_A and z_B.

In order to further demonstrate the chiral response as indicated by Fig. 3 in the main text, the Hamiltonian parameters at 1550 nm are selected to calculate the dynamics of such an unclosed path. The total propagation distance is assumed to be $Z = 243\ \mu m$. For the forwrd process, the Hamitonian starts at an infinie point, $(\beta/\kappa, \gamma/\kappa) = (-\infty, 0)$, with the initial state $|\psi(z_0)\rangle = [1,0]^T$, associated with $X_2(z_0)$ [Figs. S7(a) and S7(b)]. When the Hamiltonian evolves slowly to A, $(\beta/\kappa, \gamma/\kappa) = (0,0)$, the system state can be expressed as $|\psi(z_A)\rangle = \varepsilon X_1(z_A) + X_2(z_A)$ with $X_1(z_A) = [1,1]^T$, $X_2(z_A) = [1,-1]^T$, and ε is a small number, since X_1 is triggered as the adiabatic evolution is not strictly fulfilled. The dominant eigenmode is X_2 and the other eigenmode X_1 is infinitesimal at A. In

the interval from A to B with $\beta=0$, the system maintains the two eigenstates as $X_1=[1,1]^T$ and $X_2=[1,-1]^T$. The system state at B can be written as $|\psi(z_B)\rangle=\varepsilon\exp\left[i\int_{z_A}^{z_B}E_1(z)dz\right][1,1]^T+\exp\left[i\int_{z_A}^{z_B}E_2(z)dz\right][1,-1]^T$, according to Eq. (5) in the main text, associated with $\mathrm{Im}(E_1)=0$ and $\mathrm{Im}(E_2)>0$. The distance between A and B has been set sufficiently long to incur high loss on $[1,-1]^T$ to ensure that, $\exp\left[i\int_{z_A}^{z_B}E_2(z)dz\right]$ approaches zero, and is smaller than ε, i.e., NAJ occurs. In this situation, the system state at B is dominated by $X_1(z_B)=[1,1]^T$. In the last interval denoted by z_B-z_{end} in Fig. S7, the system keeps its dominate state as X_1, which finally evolves into $X_1(z_{end})=[1,0]^T$ at an infinie point, $(\beta/\kappa,\gamma/\kappa)=(+\infty,0)$.

For the backward process, the initial system state is $|\psi(z_{end})\rangle=[1,0]^T$, corresponding to $X_1(z_{end})$ [Figs. S7(c) and S7(d)]. The system state at B can be expressed as $|\psi(z_B)\rangle=[1,1]^T+\varepsilon[1,-1]^T$, and becomes $|\psi(z_A)\rangle=\exp\left[i\int_{z_B}^{z_A}E_1(z)dz\right][1,1]^T+\varepsilon\exp\left[i\int_{z_B}^{z_A}E_2(z)dz\right][1,-1]^T$ at A. Owning to $\mathrm{Im}(E_1)=0$, $\mathrm{Im}(E_2)>0$ and $\varepsilon<<1$, $\psi(z_A)$ can be simplified as $X_1(z_A)=[1,1]^T$. X_1 is always dominant from z_A to z_0, and finally evolves into $|\psi(z_0)\rangle=[0,1]^T$ at z_0. In the whole process from z_{end} to z_0, the system state is always dominated by X_1 and its associated eigenvalue has $\mathrm{Im}(E_1)=0$, the conversion efficiency between $X_1(z_{end})$ and $X_1(z_0)$ is close to 100%. Meanwhile, the triggered X_2 in the interval from z_{end} to z_B is nearly dissipated due to the selective coupling loss.

Using the above-mentioned method, we can obtain the dynamical evolution process as $[0,1]^T$ is used in the initial state. The final state is $[1,0]^T$ [Figs. S7(e) and S7(f)] for the forward process, and $[0,1]^T$ for the backward process owing to the occurance of NAJ [Figs. S7(g) and S7(h)]. The output state is always dominted by $[1,0]^T$ for the forward process and $[0,1]^T$ for the backward process, regardless of the initial input state.

For all different input states and evolution directions, the conversion efficiency evolving from $X_1(z_{end})$ to $X_1(z_0)$ with the initial state $[1,0]^T$ for backward process is maximum. This conversion efficiency corresponds to the chiral transmission efficiency of the output port II at the left of the device when light inputs from the port I at the right, T'_{21}, demonstrating the chiral response (Fig. 4f in the main text). We have noted in some previous studies on chiral transmission based on EP-encircling strategies, the chiral transmission efficiency is selected as the transmittance for all the different input states and encircling directions are maximum [2,3]. In our work, we have used the same definition of chiral transmission efficiency, which is consistent with the proposed open evolution trajectory. It is also worth pointing out that high-efficiency chiral transmission efficiency is guaranteed in principle as the system loss merely occurs during the NAJ process, superior over the previous schemes encircling an EP in (anti-) PT-symmetric systems with path-dependent loss. In addition, it can be seen from Figs. S7(a), S7(c), S7(e) and S7(g) that, the system evolving between A and B will not cause additional crosstalk for the output state. The crosstalk of the output state merely comes from the end of the evolution process, i.e., B to $(+\infty, 0)$ in the forward path, and A to $(-\infty, 0)$ in the backward path.

Supplementary Note 8: Fabrication and measurement setup

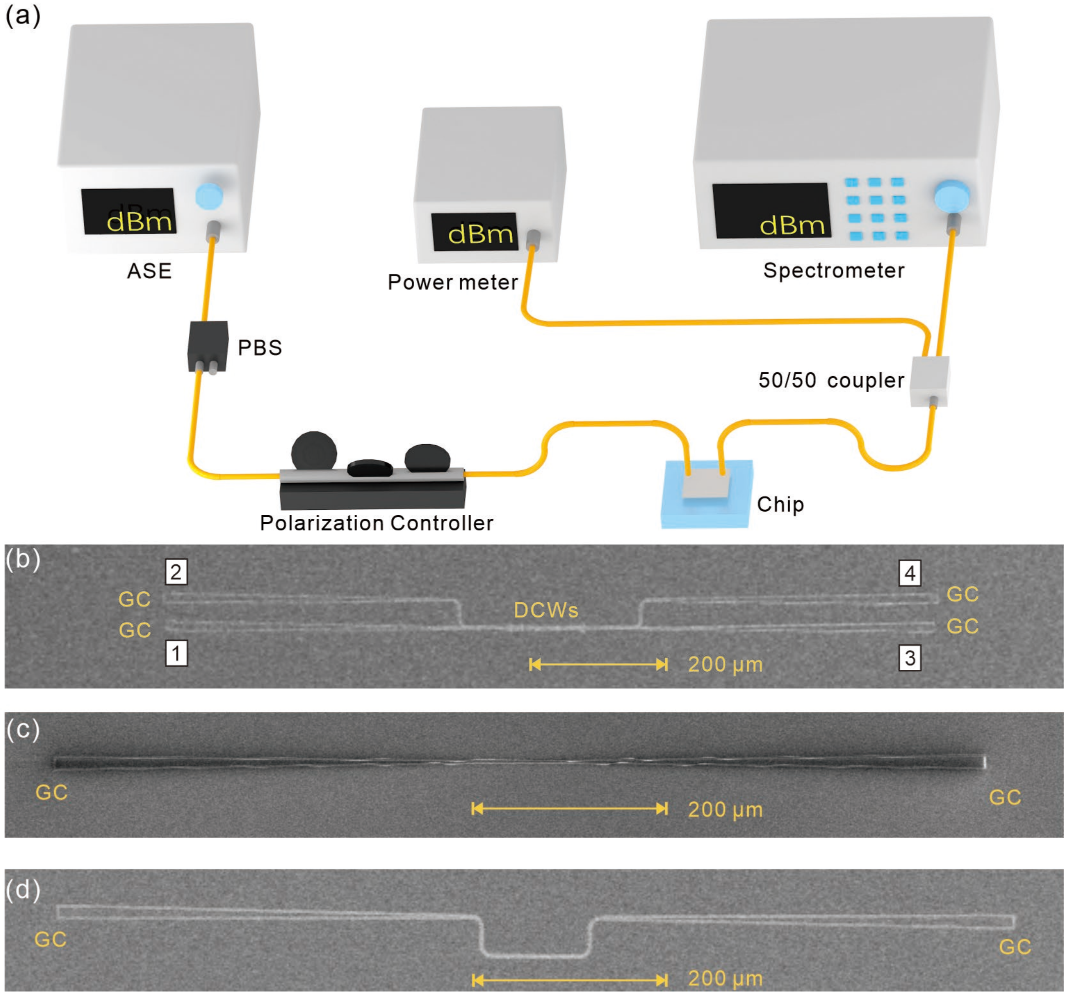

FIG. S8 | Experimental demonstration. (a) The experimental configuration. (b) The SEM image of the fabricated sample consisting of DCWs and GCs. The ports are numbered from 1 to 4. (c, d) The SEM image of the control device without DCWs, in which the waveguide is straight in (c) and bent in (d).

The sample was fabricated by a combination of electron-beam lithography and inductively coupled plasma etching. A layer of 1-μm-thick SiO_2 serves as a cover layer for symmetry of optical field and protection of the device. Figure S8(a) shows the experimental configuration for measuring the transmittance of the fabricated DCWs sample. An amplified spontaneous emission (ASE) source (OVLINK ASE-CL-20-B, a total power of 20 dBm) provides the near infrared light, and its polarization is adjusted by a polarizing beam splitter (PBS) and a polarization controller. The grating coupler

(GC) is used to couple the light from the fiber into TE_0 mode or decouple the TE_0 mode out of the silicon waveguide back into the fiber. The decoupled light will be collected by the optical power meter (PMSII-A) and a spectrometer (YOKOGAWA AQ6370C). Figures S8(b)-S8(d) show the measured SEM images of the fabricated chip samples. We can only directly record the transmission power with the power meter and spectrometer. But this does not affect how we can extract the transmission efficiencies at different ports. The optical power reaching the control waveguides is much smaller than the light source as light has to go by PBS, polarization controller and optical fiber. During this process, the major loss comes from the coupling loss between the fiber and the GC prior to the control waveguides. In the measurement, we have adjusted the coupling angle between the fiber and grating to maximize the optical power coupled into the control waveguides. This optimal coupling angle is determined if the optical power reaching the power meter is maximum, and is used for the fabricated sample consisting of DCWs and the control device without DCWs to ensure that the identical power is injected into the control waveguides. Subsequently, the transmittance at different ports can be obtained by comparing the loss differences between the fabricated sample consisting of DCWs and the control device without DCWs.

Specifically, we record the output power P_{ij}^{b}, P^{c}, and P^{d} in Fig. S8(b), S8(c) and S8(d), respectively, where P_{ij}^{b} represents the output power of the output port i when light inputs from the port j ($i, j =$ 1, 2, 3 and 4). The input power, P_{in}, is defined as the remaining optical power in the fiber after light source passes through the PBS and polarization controller. The fabricated sample consisting of DCWs and the control device without DCWs in Figs. S8(b)-S8(d) have the same GC, which incurs the identical loss on the input power. The bending radii in Figs. S8(b) and S8(d) are the same and sufficiently large, associated with near-zero loss on the input power. As a result, P^{c} is equal to P^{d}. The loss coefficient from one GC is assumed to be α^{GC}. α_{ij}^{DCW} represents the loss coefficient from the DCWs, when light inputs from the port

j and outputs from the port i ($i, j =$ 1, 2, 3 and 4). Because of the reciprocity, P_{ij}^{b} = P_{ji}^{b} must be satisfied. We can thus establish three equations associated with Figs. S8(b)-S8(d),

$$P_{ij}^{b} = P_{in} \cdot \exp(-2\alpha^{GC}) \cdot \exp(-\alpha_{ij}^{DCW}), \tag{S12}$$

$$P^{c} = P^{d} = P_{in} \cdot \exp(-2\alpha^{GC}), \tag{S13}$$

The measured transmittance T can be expressed as

$$T = \begin{cases} P_{ij}^{b} \big/ P^{c} & (i, j \ = \ 2,\ 4 \text{ and } i \neq j) \\ P_{ij}^{b} \big/ P^{d} & (i, j \ = \ 1,\ 3 \text{ and } i \neq j) \\ P_{ij}^{b} \big/ \sqrt{P^{c} \cdot P^{d}} & (i, j \ = \ 1,\ 4 \text{ or } i, j \ = \ 2,\ 3 \text{ and } i \neq j) \end{cases}, \tag{S14}$$

Incorporating Eqs. (S12-S13) into Eq. (S14), yielding

$$T = \exp(-\alpha_{ij}^{DCW}), \tag{S15}$$

Therefore, the measured transmittance in Eq. (S14) accurately reflects the transmission characteristics of the DCWs. The measured power at different ports with the optical spectrometer is shown in Figs. S9. The measured transmittance spectra shown in Fig. 4 of the main text and supplementary Fig.S10 are with

$$\begin{aligned} T_{21}' &= \exp(-\alpha_{23}^{DCW}), & T_{21} &= \exp(-\alpha_{41}^{DCW}), \\ T_{11}' &= \exp(-\alpha_{13}^{DCW}), & T_{11} &= \exp(-\alpha_{31}^{DCW}), \\ T_{12}' &= \exp(-\alpha_{14}^{DCW}), & T_{12} &= \exp(-\alpha_{32}^{DCW}), \\ T_{22}' &= \exp(-\alpha_{24}^{DCW}), & T_{22} &= \exp(-\alpha_{42}^{DCW}). \end{aligned} \tag{S16}$$

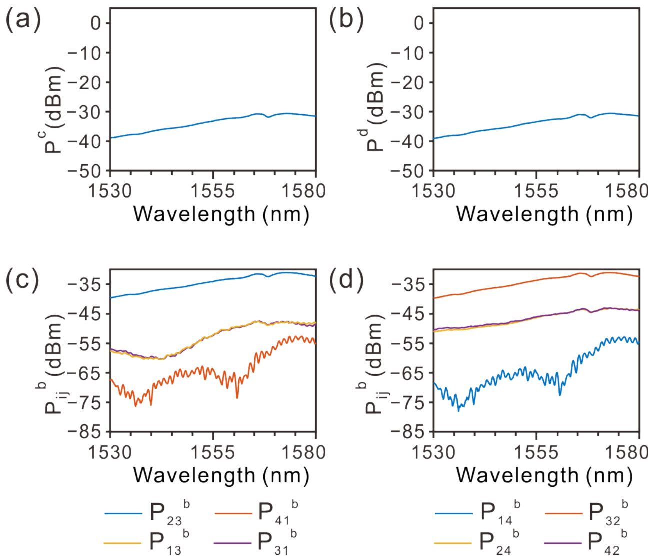

FIG. S9 | The measured optical power at different ports. (a) The power through two GCs in Fig. S8(c). (b) The power through two GCs with two bending waveguides in Fig. S8(d). (c, d) The power in Fig. S8(b) as the DCWs is involved.

Supplementary Note 9: Simulated and experimental results with $[0,1]^T$ input

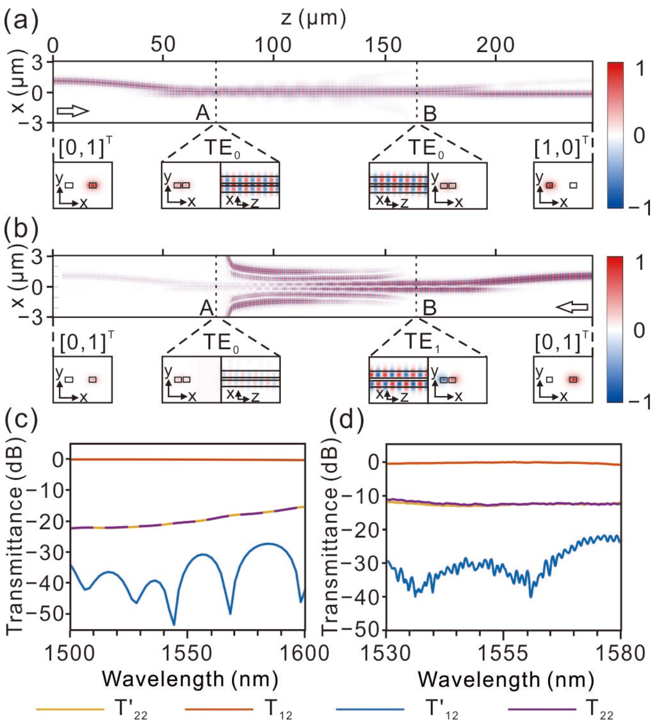

FIG. S10 | Simulated and experimental results. (a, b) Simulated field distributions of E_x at 1550 nm when port II is excited from the left (a) and right (b), respectively. (a) Simulated transmittance spectra at the output port over the wavelength range of 1500-1600 nm. (b) Measured transmittance spectra at the output port over the wavelength range of 1530-1580 nm.

Reference

1 Abitan, H., Bohr, H. & Buchhave, P. Correction to the Beer-Lambert-Bouguer law for optical absorption. *Appl. Opt.* **47**, 5354-5357, doi:10.1364/AO.47.005354 (2008).

2 Shu, X. *et al.* Fast encirclement of an exceptional point for highly efficient and compact chiral mode converters. *Nat. Commun.* **13**, 2123, doi:10.1038/s41467-022-29777-5 (2022).

3 Liu, Q. *et al.* Efficient Mode Transfer on a Compact Silicon Chip by Encircling Moving Exceptional Points. *Phys. Rev. Lett.* **124**, 153903, doi:10.1103/PhysRevLett.124.153903 (2020).
